# Ozone, drought, photosynthesis, and yield: How would soybean face a Mad Max scenario?

**DOI:** 10.1093/plphys/kiaf368

**Published:** 2025-08-20

**Authors:** Marcella Alves Teixeira

**Affiliations:** Department of Plant Pathology, Washington State University, Pullman, WA 99163, USA

Most of us have watched movies like Mad Max, set in a world with severe conditions and in which water is a luxurious commodity. Have you ever wondered about cropping in such scenarios? Although those movies are extreme, science shows that ozone concentrations significantly increased in the past century, and some models using high carbon emission scenarios expect ozone concentrations to continue to increase for the next 50 years (Eyring et al. 2013; Karagodin-Doyennel et al. 2023). Ozone is a problem because it can diffuse through plants stomata and be converted into other reactive oxygen species, resulting in lower photosynthetic rates, early senescence, and, ultimately, reduced productivity (Fiscus et al. 2005; Ainsworth 2017). Additionally, changes in the climate are also expected to increase the frequency and severity of drought events (Jin et al. 2018; IPCC 2021), the most limiting factor for crop photosynthesis and yield (Boyer 1982).

What happens when both high ozone concentration and drought occur at the same time in the field? One theory is that since plants reduce stomatal aperture during drought, this would lead to protection against elevated ozone. The high ozone concentration can also lead to tissue damage, interfering with stomatal closure. In this issue of *Plant Physiology*, Martin et al. (2025) investigated these hypotheses using a large-scale free air concentration enrichment system (Fig. 1A). They carried out field-based experiments to expose soybean to ozone pollution during 3 growing seasons and capture 40% of rainfall, significantly reducing soil moisture content throughout these growing seasons. Looking at the isolated drought effect (Fig. 1B), they observed that capturing 40% of the rainfall only resulted in reduced photosynthetic capacity and seed yields in one growing season, the one with the smaller rain volume. This reduced soil moisture resulted in increased ABA content (Fig. 1B). Although their results showed no evidence that drought prevented damage to photosynthetic capacity, Martin et al. (2025) observed a positive correlation between CO_2_ assimilation and soil moisture specifically when there was no elevated ozone treatment.

**Figure 1.**
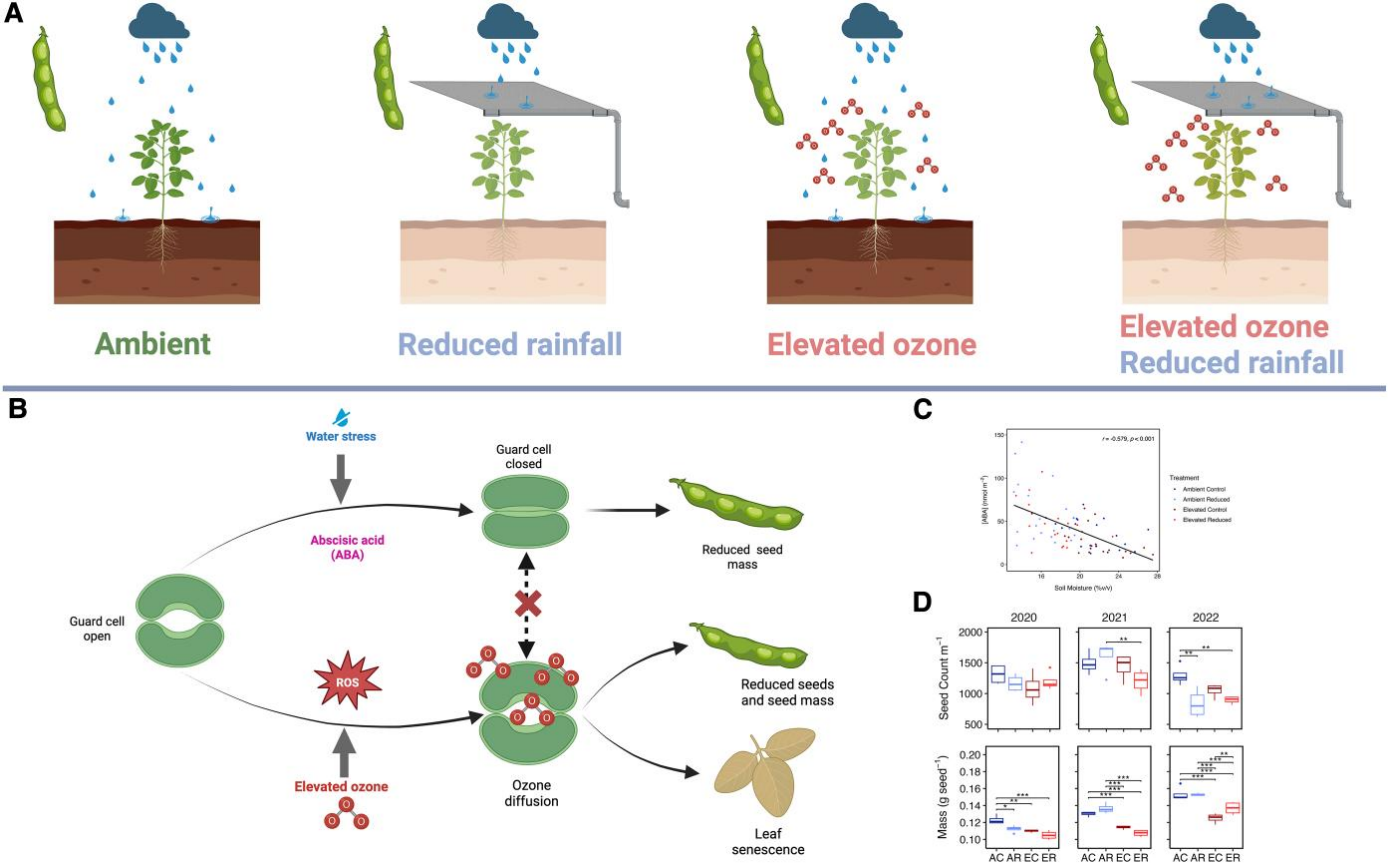
Elevated ozone impacts are not mitigated by soil drying. The 4 treatments used in the SoyFACE setup are: ambient—with ambient ozone and control precipitation (AC), reduced rainfall—with ambient ozone and reduced precipitation (AR), elevated ozone—with elevated ozone and control precipitation (EC), and elevated ozone/reduced rainfall—with elevated ozone and reduced precipitation (ER). **A)** The 4 treatments show precipitation collection and ozone application. Seeds mass and numbers are also represented in the pods located on the left side of each treatment. **B)** Representation of stomatal response to water stress and elevated ozone treatments. Capturing rainfall leads to water stress and changes in abscisic acid (ABA) accumulation, resulting in stomatal closure and reduced seed mass. Elevated ozone treatment leads to increased diffusion into leaves, resulting in reduced seed number and mass, besides leaf senescence. The double treatment, with both rainfall capture and elevated ozone, showed that stomatal closure caused by drought does not limit the damage caused by increased ozone. **C)** Correlation between leaf ABA content and soil moisture (%v/v) measured at 20-cm depth in soybeans subjected to the 4 treatments. **D)** Seed number per 1-m row and individual seed mass in soybeans subjected to the 4 treatments in 2020, 2021, and 2022. Adapted from Martin et al. 2025. Created in BioRender. Teixeira, M. (2025) https://BioRender.com/ygwy8ij.

When Martin et al. (2025) treated soybean with elevated ozone, there were no significant effects on ABA or H_2_O_2_. Therefore, these results add to the claim that H_2_O_2_ level is an inconsistent indicator of ozone and drought stress, as observed by others (Cheeseman 2009). Contrary to previous publications, Martin et al. (2025) showed that reduced soil moisture did not decrease negative effects of ozone on photosynthetic capacity and yield, as shown by evaluation of the maximum rates of carboxylation of Rubisco (V_cmax_), electron transport (J_max_), yield, and photosynthetic capacity. Photosynthetic capacity was reduced by elevated ozone, especially in older leaves exposed to a higher dose of ozone.

Ultimately, in the sci-fi movies and in real life, the great concern about these stresses is the yield reduction. Martin et al. (2025) show that elevated ozone leads to reduced seed number and mass by 25% across growing seasons. Surprisingly, drought only impacted yield in the year with lower rainfall, when the authors observed reduced number of seeds but no changes in individual seed mass (Fig. 1B).

Martin et al. (2025) challenged the theory that drought-induced stomatal closure can interfere with ozone influx into soybean leaves, showing that elevated ozone is damaging even when there is drought (Fig. 1B). The authors show that a “Mad max scenario,” with the combination of elevated ozone and drought, is enough to reduce V_cmax_, integrated carbon assimilation, and yield. It will be interesting to see similar field investigations exploring the impacts of stress timing, plants’ potential to recover from drought, and ozone impact on distinct tissues. Future investigations will allow researchers to characterize plants’ physiological responses to abiotic stress combinations and length and will serve as guidelines for breeding programs aiming at climate resilience.

## Data Availability

No new data were generated or analysed in support of this research.
